# Incremental value of dedicated [^201^Tl] cadmium-zinc-telluride thallium SPECT over rest/stress [^99m^Tc] technetium sestamibi SPECT MPI: a comparative analysis of fixed perfusion defects

**DOI:** 10.3389/fcvm.2026.1901315

**Published:** 2026-10-02

**Authors:** Phattaraphorn Teungsang, Methawee Laohamethanee, Napat Kitkhuandee, Thanapol Kliteneam, Daris Theerakulpisut

**Affiliations:** 1 Faculty of Medicine, Khon Kaen University, Khon Kaen, Thailand; 2 Division of Nuclear Medicine, Department of Radiology, Faculty of Medicine, Khon Kaen University, Khon Kaen, Thailand

**Keywords:** cardiovascular imaging, myocardial perfusion abnormality, perfusion defect quantification, radionuclide imaging, sestamibi myocardial imaging, SPECT MPI, thallium (I) chloride

## Abstract

**Background:**

Rest/stress [^99m^Tc]technetium sestamibi single-photon emission computed tomography myocardial perfusion imaging (SPECT MPI) is a mainstay for evaluating coronary artery disease. However, fixed defects on standard MPI present a well-documented clinical challenge, as they can represent either non-viable scars or viable hibernating myocardium. We aimed to examine the incremental value and rate of diagnostic reclassification provided by performing subsequent dedicated cadmium-zinc-telluride (CZT) [^201^Tl]thallium myocardial SPECT in patients presenting with fixed defects on standard sestamibi MPI.

**Methods:**

In this retrospective study, we analyzed 174 patients—totaling 2,958 myocardial segments—who underwent both protocols. Intermodality correlation was assessed, and the rates of segmental and patient-level reclassification (segments converting from <50% maximum uptake on sestamibi to ≥50% maximum uptake on thallium) were quantified.

**Results:**

While a moderately high correlation was observed between the baseline uptake scores of the two modalities (Spearman's r = 0.817 for rest sestamibi vs. thallium), thallium imaging provided substantial additional information, with 48.0% of segments classified as fixed defects on sestamibi SPECT demonstrating improved uptake on thallium SPECT. At the patient level, 78.9% of individuals who had fixed defects on the initial sestamibi SPECT had at least one segment reclassified. Receiver operating characteristic analysis showed that a rest sestamibi uptake score of <20% predicted a lack of reclassification with 99% specificity, although with low sensitivity (12%).

**Conclusion:**

In a selected cohort of patients presenting with fixed defects on standard rest/stress sestamibi SPECT MPI, supplementary imaging with thallium myocardial CZT SPECT provided incremental data, altering the clinical interpretation in nearly 80% of patients. However, in the absence of an independent viability reference standard, this finding represents intertracer diagnostic reclassification rather than proven functional recovery. A rest sestamibi uptake of <20% demonstrated high specificity for non-reclassification; however, this cutoff should be interpreted cautiously as a potential gatekeeping finding when considering downstream testing rather than a rigid rule for omitting further myocardial viability evaluation.

## 1 Introduction

Coronary artery disease is the most common cardiovascular disease, affecting 315 million people globally (1), and is the leading cause of death worldwide (2). Among a myriad of non-invasive diagnostic tests for patients with chronic coronary artery disease, the nuclear medicine imaging modality, single-photon emission computed tomography myocardial perfusion imaging (SPECT MPI), remains one of the leading modalities used to detect the presence and extent of myocardial ischemia. It provides valuable prognostic information for determining the risk of major cardiovascular events and, in turn, helps select the appropriate treatment strategy (3). Rest/stress SPECT MPI is one of the most commonly used imaging protocols. Reversible defects (defects in the stress SPECT which improves in the rest SPECT) indicate myocardial ischemia, while fixed defects (spatially congruent defects in both the stress and rest SPECT) could indicate non-viable myocardial scarring. However [^99m^Tc]Technetium sestamibi SPECT MPI has been shown to underestimate myocardial viability, with fixed defects showing signs of viability in other imaging modalities (4–6). Various imaging modalities are available for evaluation of myocardial viability, including dobutamine stress echocardiography, cardiac magnetic resonance imaging (CMR), [^18^F]fluorodeoxyglucose (FDG) Positron emission tomography (PET) PET, and [^201^Tl]thallium myocardial SPECT (7, 8). Although [^18^F]FDG PET and CMR are recognized as highly sensitive benchmarks for myocardial viability, [^201^Tl]thallium SPECT remains a widely accessible alternative in centers lacking PET infrastructure (9). In our practice environment, [^18^F]FDG PET is not covered by national health reimbursement schemes for cardiac indications, and local availability to CMR is constrained by long waiting times. Consequently, sequential [^201^Tl]thallium SPECT imaging following initial rest/stress [^99m^Tc]Technetium sestamibi SPECT MPI in patients who have fixed defects provides a timely and pragmatic clinical surrogate for viability evaluation.

Although the fundamental physiological concept that [^201^Tl]thallium redistribution can identify viability in seemingly fixed [^99m^Tc]Technetium sestamibi defects is well established, its clinical implementation has historically faced technical constraints. On conventional Anger cameras, late delayed imaging, such as, 24-h [^201^Tl]thallium imaging, is limited by low count statistics and prolonged acquisition times, which could increase patient motion artifacts. In contemporary nuclear cardiology, solid-state cadmium-zinc-telluride (CZT) camera technology acts as a critical enabler that overcomes these physical hardware limitations. With higher photon sensitivity and spatial resolution, modern CZT systems enable preservation of quantitative segmental uptake accuracy even under count-reduced or shortened acquisition protocols (10).

Rather than reevaluating tracer kinetics or asserting superior physical resolution compared with older hardware, this study reports our real-world clinical experience and quantifies the incremental diagnostic yield provided by adding a dedicated [^201^Tl]thallium CZT SPECT workflow to routine [^99m^Tc]Technetium sestamibi SPECT MPI. In particular, we demonstrate the practical extent of intermodality defect reclassification achievable when utilizing CZT hardware in everyday clinical practice.

## 2 Materials and methods

### 2.1 Patients

This single-center, retrospective, cross-sectional study was approved by the Khon Kaen University Ethics Committee for Human Research (reference number HE671083). Due to its retrospective nature, requirements for obtaining informed consent were waived by the ethics committee. Access to patient data was approved by the hospital director. Patients aged 18 years or above with known or suspected coronary artery disease who underwent both rest/stress [^99m^Tc]Tc-sestamibi SPECT MPI and [^201^Tl]thallium myocardial SPECT at the Division of Nuclear Medicine, Department of Radiology, Srinagarind Hospital, Faculty of Medicine, Khon Kaen University, between 1 January 2013 and 31 December 2022 were included. Cases in which the two imaging studies were performed more than 3 months apart and cases with significant imaging artifacts that interfered with interpretation and quantitative measurements were excluded.

### 2.2 Rest/stress [^99m^Tc]technetium sestamibi SPECT MPI protocol

All patients underwent a one-day rest/stress adenosine [^99m^Tc]Tc-sestamibi SPECT MPI protocol. Beta-blockers, calcium channel blockers, and oral nitrates were withheld at the attending physician's discretion. Caffeine-containing beverages and foods were not allowed for 24 h, and patients were instructed to withhold solid meals for at least 3 h before imaging. For the rest study, 7 mCi of [^99m^Tc]Tc-sestamibi was intravenously injected, and rest SPECT was acquired 45–60 min later. The stress procedure commenced on the same day after completion of the rest imaging. Patients were intravenously administered adenosine at a rate of 140 mcg/kg/min for 6 min. A 25 mCi dose of [^99m^Tc]Tc-sestamibi was injected at the third minute of adenosine infusion. Electrocardiography (ECG) was performed throughout the procedure. Stress SPECT was acquired 30–60 min after the stress dose injection. Imaging was performed using a Discovery NM530 dedicated cardiac CZT SPECT (GE Healthcare, Chicago, IL, USA) (GE Healthcare, IL, USA). ECG-gated cardiac SPECT imaging was acquired for 6 and 3 min for the rest SPECT and stress SPECT, respectively. The energy peak was set at 140.5 keV ± 10%, and a 256 × 256 imaging matrix was used.

### 2.3 [^201^Tl]thallium myocardial SPECT protocol

Patients were selected to undergo [^201^Tl]thallium myocardial SPECT if the rest/stress [^99m^Tc]Tc-sestamibi SPECT MPI demonstrated a fixed perfusion defect. Two clinical imaging pathways were utilized, determined primarily by camera slot availability and radiotracer procurement logistics, as follows.

#### 2.3.1 Direct 24-h delayed protocol

If an imaging slot was available on the following day, patients were injected with 3 mCi of [^201^Tl]thallous chloride immediately after the completion of the sestamibi study and returned 24 h later for delayed imaging. A total of 112 patients (64.4%) underwent this protocol.

#### 2.3.2 Scheduled rest redistribution protocol

If next-day camera availability was constrained or tracer delivery required advance scheduling, patients were scheduled for a formal rest redistribution [^201^Tl]thallium myocardial SPECT study at the next earliest available date. This involved an initial 2.5 mCi [^201^Tl]thallous chloride injection with 1- and 4-h imaging, followed by a 1.5 mCi booster reinjection and 24-h imaging if severe hypoperfusion (<50% maximum uptake) persisted in the 4-h images. A total of 62 patients (35.6%) underwent this protocol.

ECG-gated SPECT imaging was performed using the same Discovery NM530 dedicated cardiac CZT SPECT system (GE Healthcare, IL, USA) with an acquisition time of 6 min. The energy peaks were set at 70.0 keV (−14% to +23%) and 167.0 keV ± 9%. An imaging matrix of 256 × 256 was used.

### 2.4 Image analysis and interpretation

All images were analyzed and interpreted by a nuclear medicine physician with 15 years of experience in nuclear cardiology (DT). Raw images were analyzed using ECToolbox and Myovation software included in the Xeleris 3 software suite provided by the machine vendor. Images were displayed as a 17-segment polar map according to the American Society of Nuclear Cardiology guidelines (11), together with uptake scores representing the percentage of average uptake compared with the maximum uptake in the myocardium. The uptake score for each individual segment was recorded. For cases where [^201^Tl]thallium myocardial SPECT was performed at more than one time point, the highest uptake score among all of the time points was used. A segment with an uptake of ≥50% of maximum myocardial activity was considered viable, whereas a segment with an uptake of <50% of maximum activity was considered non-viable.

### 2.5 Statistical analysis

Patient demographics are presented using descriptive statistics. Continuous data are summarized as mean and standard deviation. Categorical data are expressed as counts and percentages. Correlations between uptake on rest and stress [^99m^Tc]Tc-sestamibi SPECT MPI with [^201^Tl]thallium myocardial SPECT were tested using Spearman's rank correlation coefficient, and agreement was evaluated using the intraclass correlation coefficient (ICC). As an exploratory analysis, receiver operating characteristic (ROC) curve analysis was performed to determine if the uptake score on rest or stress SPECT MPI could predict [^201^Tl]thallium myocardial SPECT outcomes and to identify potential cutoff values for rest or stress [^99m^Tc]Tc-sestamibi SPECT MPI uptake score that could reliably predict if a segment will demonstrate uptake of <50% on [^201^Tl]thallium myocardial SPECT. Accompanying 95% confidence intervals (95% CI) were reported where appropriate. All statistical analyses were conducted using R version 4.4.2 (R Core Team, 2024) and RStudio version 2024.12.0+467. Key statistical packages included tidyverse version 2.0.0, irr version 0.84.1, and pROC version 1.18.5.

## 3 Results

A total of 174 patients were included, comprising 2,958 myocardial segments. Patient characteristics are presented in Table 1. The average age was 65.0 years, and the study population consisted of 77.3% men. The cohort had average body habitus, with an average weight of 64.0 kg and average height of 162.1 cm. [^201^Tl]thallium myocardial SPECT revealed that 32.8% of patients had ≥50% maximum uptake in all segments, whereas 67.2% of patients had at least one segment with <50% maximum uptake.

**Table 1:** Patient characteristics.

Characteristic	Value	SD/Percent
Age (years, mean ± SD)	65.0	±13.6
Sex		
Male	134	77.0%
Female	40	23.0%
Body weight (kg, mean ± SD)	64.0	± 14.4
Height (cm, mean ± SD)	162.1	± 7.0
[^201^Tl]thallium myocardial SPECT results		
All segments have ≥50% maximum uptake	57	32.8%
At least one segment has <50% maximum uptake	117	67.2%

There was moderate agreement between stress [^99m^Tc]Tc-sestamibi SPECT MPI and [^201^Tl]thallium myocardial SPECT (Cohen's kappa 0.491, 95% CI 0.454–0.529, *p* < 0.001), and a slightly better agreement between rest [^99m^Tc]Tc-sestamibi SPECT MPI and [^201^Tl]thallium myocardial SPECT (Cohen's kappa = 0.550 (95% CI. 0.512–0.588; *p*-value, < 0.001), as shown in Tables 2, 3.

**Table 2:** Agreement between stress [^99m^Tc] technetium sestamibi SPECT MPI and [^201^Tl] thallium myocardial SPECT.

Agreement		[^201^Tl]thallium myocardial SPECT	
		<50% maximum uptake	≥50% maximum uptake
Stress [^99m^Tc]Tc-sestamibi SPECT MPI	<50% maximum uptake	316	414
	≥50% maximum uptake	52	2,176

Cohen's kappa = 0.491 (0.454–0.529; *p*-value < 0.001).

**Table 3:** Agreement between rest [^99m^Tc] technetium sestamibi SPECT MPI and [^201^Tl] thallium myocardial SPECT.

Agreement		[^201^Tl]thallium myocardial SPECT	
		<50% maximum uptake	≥50% maximum uptake
Rest [^99m^Tc]Tc-sestamibi SPECT MPI	<50% maximum uptake	326	353
	≥50% maximum uptake	42	2,237

Cohen's kappa = 0.550 (0.512–0.588; *p*-value < 0.001).

There was significant correlation and agreement between stress and rest [^99m^Tc]Tc-sestamibi SPECT MPI uptake scores and [^201^Tl]thallium myocardial SPECT uptake scores. For stress [^99m^Tc]Tc-sestamibi SPECT MPI, Spearman's rank correlation coefficient was 0.790 (95% CI 0.776–0.803, *p* < 0.001). The ICC was 0.781 (95% CI 0.767–0.796, *p* < 0.001). For rest [^99m^Tc]Tc-sestamibi SPECT MPI the Spearman's rank correlation coefficient was 0.817 (95% CI 0.805–0.829, *p* < 0.001) and the ICC was 0.812 (95% CI 0.800–0.824, *p* < 0.001), as shown in Table 4.

**Table 4:** Correlation and agreement of uptake scores between rest and stress [^99m^Tc]Tc-sestamibi SPECT MPI with [^201^Tl]thallium myocardial SPECT.

Measure	Comparator with [^201^Tl] thallium myocardial SPECT	Value	95% CI	*p*-value
Spearman's rank correlation coefficient	Stress sestamibi SPECT MPI	0.790	0.776–0.803	<0.001
	Rest sestamibi SPECT MPI	0.817	0.805–0.829	<0.001
Intraclass correlation coefficient	Stress sestamibi SPECT MPI	0.781	0.767–0.796	<0.001
	Rest sestamibi SPECT MPI	0.812	0.800–0.824	<0.001

CI, confidence interval.

To determine the impact of [^201^Tl]thallium myocardial SPECT on reclassifying patients with fixed defects suspected of being non-viable from the rest/stress [^99m^Tc]Tc-sestamibi SPECT MPI, subgroup analysis was performed in patients whose rest and stress [^99m^Tc]Tc-sestamibi SPECT MPI had at least one segment with uptake <50%. Segment-wise, among the 587 myocardial segments which had < 50% maximum uptake on both rest and stress [^99m^Tc]Tc-sestamibi SPECT MPI, 282 (48.0%) converted to having ≥50% maximum uptake on [^201^Tl]thallium myocardial SPECT. Patient-wise, among the 152 patients who had at least one segment with < 50% maximum uptake on both rest and stress sestamibi SPECT MPI, 120 (78.9%) had at least one segment which was converted to ≥50% maximum uptake in thallium myocardial SPECT, as shown in Table 5. An example of a case in which thallium myocardial SPECT resulted in reclassification of defects is shown in Figure 1.

**Table 5:** Proportion of patients and myocardial segments which converted from <50% maximum uptake in rest/stress SPECT MPI to ≥50% maximum uptake in thallium myocardial SPECT.

Analysis approach	Thallium myocardial SPECT results	Number	Percent
Segment-wise	Segments that remained < 50% maximum uptake	305	52.0
	Segments that converted to ≥50% maximum uptake	282	48.0
Patient-wise	Patients whose results remained unchanged	32	21.1
	Patients with at least one segment converted to ≥50% maximum uptake	120	78.9

**Figure 1:**
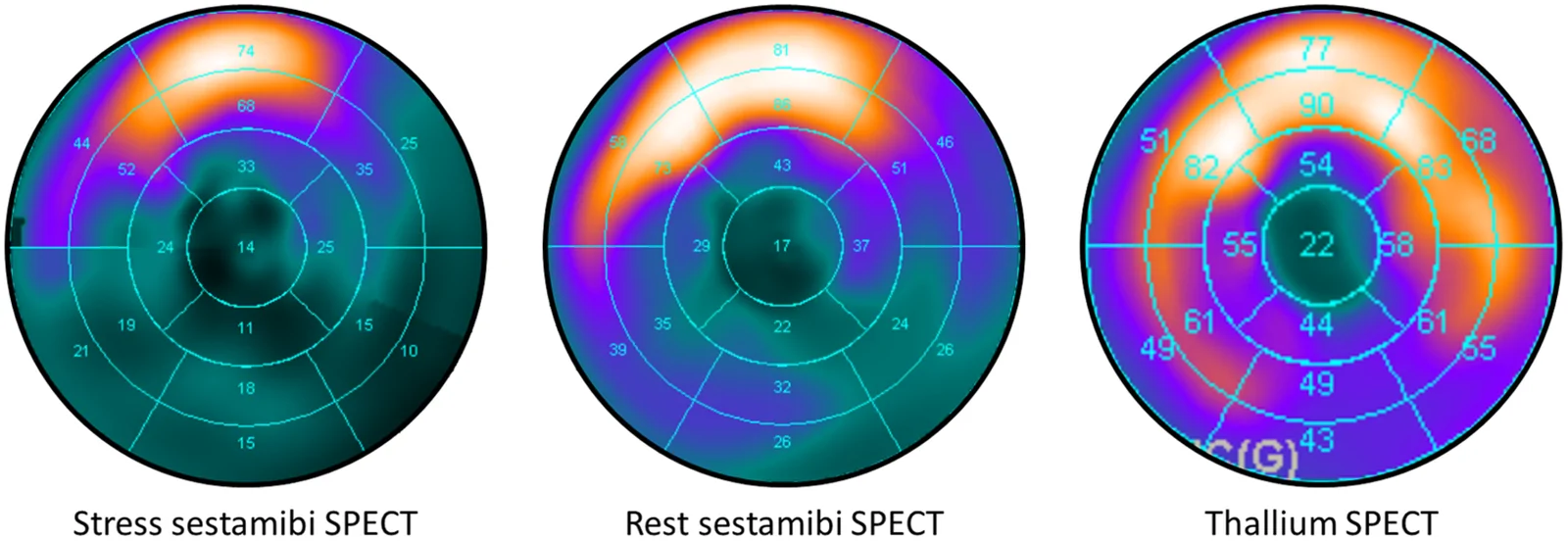
An example case depicting a large, fixed defect at the apex inferior, inferoseptal, and lateral wall of the left ventricle having uptake scores of <50% maximum uptake in both the rest and stress sestamibi SPECT MPI, involving 12 myocardial segments. However, there is significant improvement in the uptake scores in the thallium SPECT with only five segments having scores remaining <50% maximum uptake, with seven segments converting to ≥50% maximum uptake.

To address concerns regarding whether the choice of thallium imaging protocol could impact the study findings, subgroup analysis comparing the direct 24-h delayed protocol (*n* = 112) and scheduled rest redistribution protocol (*n* = 62) was performed, which revealed consistent findings across both protocols (Supplementary Tables S1–S3) in terms of patient characteristics and study effects. Segment-level conversion from <50% to ≥50% maximum uptake occurred in 48.9% of segments in the direct 24-h delayed protocol and 46.9% in the scheduled rest redistribution protocol. At a patient level, 78.6% of patients in the direct 24-h delayed protocol and 79.6% in the scheduled rest redistribution protocol had at least one reclassified segment. Intermodality correlation and agreement, as determined by the Spearman's rank correlation coefficient, the intraclass correlation coefficient, and Cohen's kappa, were similar across the two subgroups.

ROC curve analysis revealed that, in segments with fixed defects—with uptake scores of <50% in both the rest and stress [^99m^Tc]Tc-sestamibi SPECT MPI—the uptake score of stress [^99m^Tc]Tc-sestamibi SPECT MPI and rest [^99m^Tc]Tc-sestamibi SPECT MPI had only a fair degree of predictiveness for predicting “converters,” defined as having an uptake score of ≥ 50% maximum uptake on [^201^Tl]thallium myocardial SPECT, with area under the curves (AUC) of 0.747 (95% CI. 0.708–0.787) and 0.779 (95% CI. 0.742–0.816), respectively, as shown in Figure 2. As the AUC for rest [^99m^Tc]Tc-sestamibi SPECT MPI was higher than for stress [^99m^Tc]Tc-sestamibi SPECT MPI, further exploration was performed to determine whether there was an appropriate cutoff for the rest [^99m^Tc]Tc-sestamibi SPECT MPI uptake score which could predict <50% maximum uptake on [^201^Tl]thallium myocardial SPECT, thus negating the need for additional thallium imaging. The analysis revealed that a cutoff of 20% maximum uptake for the rest uptake score had a specificity of 99% for persistent deficient uptake of <50% maximum uptake on [^201^Tl]thallium myocardial SPECT, but with a sensitivity of only 12%. For a more conservative cutoff of 30% uptake, the specificity for <50% maximum uptake on [^201^Tl]thallium myocardial SPECT was slightly less, at 93%, but with an increased sensitivity of 36%.

**Figure 2:**
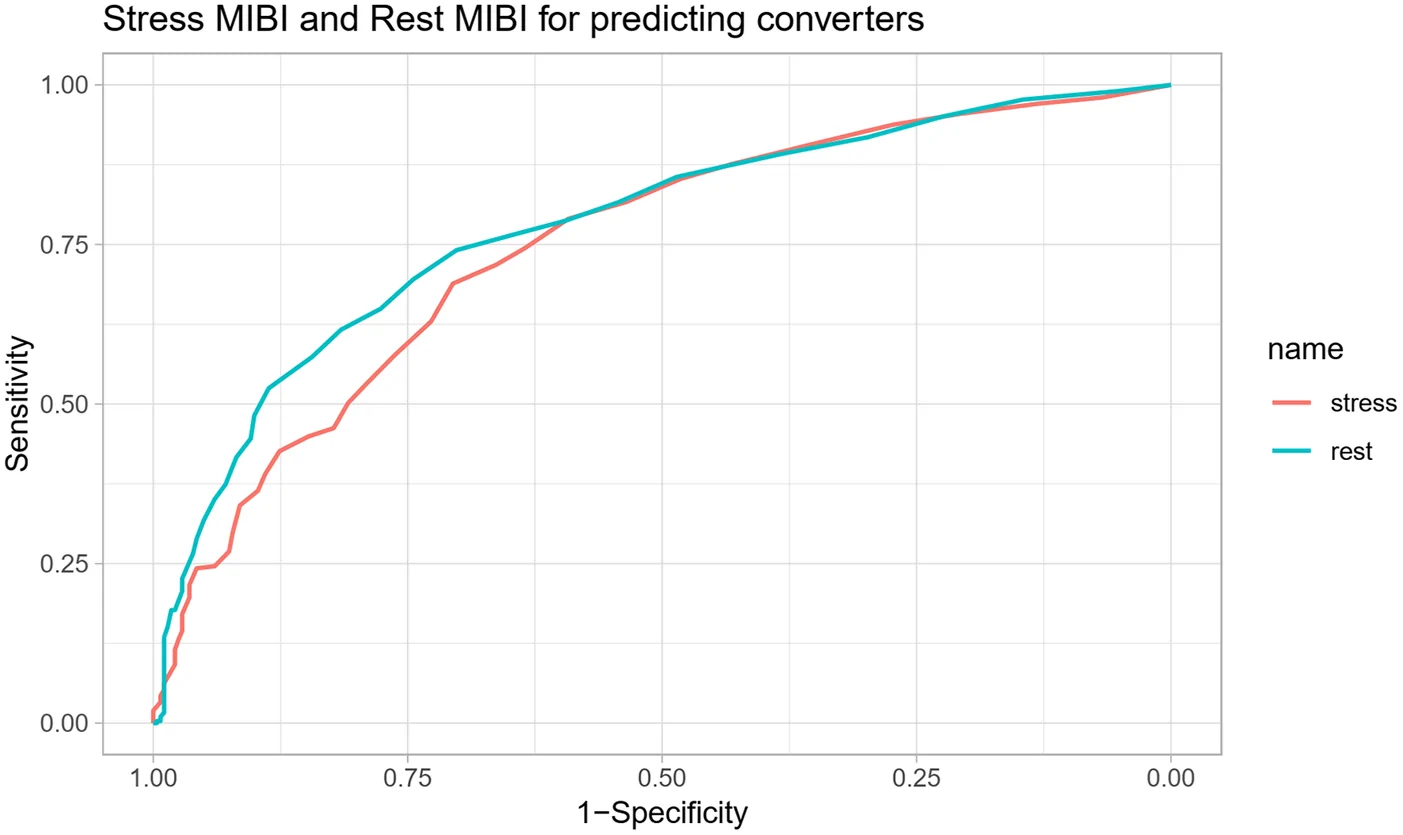
Receiver operating characteristic curves of the stress and rest [^99m^Tc]Tc-sestamibi SPECT MPI uptake scores for distinguishing <50% maximum uptake vs. ≥50% maximum uptake on [^201^Tl]thallium myocardial SPECT, among myocardial segments which had fixed defects with <50% maximum uptake in both rest and stress SPECT MPI.

## 4 Discussion

This study aimed to evaluate the intermodality correlation and clinical reclassification rate between rest/stress [^99m^Tc]Tc-sestamibi SPECT MPI and subsequent [^201^Tl]thallium myocardial SPECT using dedicated CZT cardiac SPECT.

By directly comparing these two protocols in a large patient cohort, we sought to determine the level of segmental agreement, the proportion of patients whose initial imaging results were functionally reclassified by [^201^Tl]thallium myocardial SPECT, and whether a specific [^99m^Tc]Tc-sestamibi SPECT MPI uptake score threshold exists that could safely serve as a gatekeeping cutoff to optimize the diagnostic workflow. Our findings provide important clinical insights into how these modalities diverge and outline how existing CZT technology can be utilized effectively without duplicating unnecessary testing. In this analysis, an uptake score threshold of ≥50% of maximum myocardial activity was operationally used to suggest preserved tissue integrity, a threshold well supported by established nuclear cardiology literature demonstrating a high probability of functional recovery after myocardial revascularization (12, 13).

Although we found moderately high correlation and agreement between rest and stress [^99m^Tc]Tc-sestamibi SPECT MPI and [^201^Tl]thallium myocardial SPECT in terms of uptake scores (Spearman's r = 0.817 for rest sestamibi), a significant discrepancy was observed when applying the standard 50% of maximum uptake threshold to determine diagnostic categories. In particular, 48% of segments demonstrating deficient uptake (<50% of maximum uptake) on rest/stress [^99m^Tc]Tc-sestamibi SPECT MPI were reclassified as having preserved uptake (≥50% of maximum uptake) on [^201^Tl]thallium myocardial SPECT. Furthermore, 78.9% of patients with fixed defect(s) on rest/stress [^99m^Tc]Tc-sestamibi SPECT MPI had at least one segment reclassified to preserved uptake on [^201^Tl]thallium myocardial SPECT.

Rather than signaling a flaw in either modality, this distinct intermodality discordance highlights the additional incremental information provided by subsequent [^201^Tl]thallium imaging when evaluating seemingly fixed defects. Crucially, this finding reflects a rate of tracer diagnostic reclassification rather than a direct, benchmarked confirmation of true myocardial viability or post-revascularization functional recovery. Without confirmatory testing with [18F]FDG PET or late gadolinium enhancement cardiac MRI, segmental conversion from <50% to ≥50% maximum uptake must be interpreted strictly as intermodality tracer uptake reclassification between [^99m^Tc]Tc-sestamibi and [^201^Tl]thallium SPECT, rather than benchmarking against an absolute reference standard for myocardial viability.

The primary physiological mechanism driving this diagnostic divergence lies in the fundamentally different pharmacokinetics of the two radiopharmaceuticals. [^201^Tl]thallium, a potassium analog that enters the cells via the active Na^+^/K^+^-ATPase pump, freely redistributes into ischemic but viable myocardium over time, allowing identification of regions with preserved cell membrane integrity and preserved cellular function (14).

[^99m^Tc]Tc-sestamibi, on the other hand, behaves as a cellular perfusion marker that binds tightly to mitochondrial membranes and exhibits minimal redistribution over time; therefore, it does not reflect the dynamic changes in myocardial perfusion and metabolism as effectively (15). Consequently, in regions of severe resting hypoperfusion, it may not adequately reflect underlying cellular viability, as documented in a previous study (4).

Concerns regarding potential bias caused by utilizing different [^201^Tl]thallium SPECT protocols were addressed by performing subgroup analyses, which showed that reclassification rates were virtually identical between patients undergoing direct 24-h delayed protocol and those undergoing the scheduled rest redistribution protocol (48.9% vs. 46.9% at the segment level; 78.6% vs. 79.6% at the patient level; Supplementary Table S3). This demonstrates that the two clinical workflow pathways yielded consistent reclassification outcomes.

The consistency of both pathways may be explained by the technical advantages of dedicated CZT SPECT technology. The enhanced photon sensitivity and high count rate of solid-state CZT detectors allow for fast 6-min acquisitions while maintaining image counting statistics, even for the 24-h delayed imaging, when remaining radiotracer activity is reduced.

Although our ROC analysis identified a rest [^99mTc^]Tc-sestamibi SPECT MPI uptake threshold of <20% as a strong predictor of fixed deficiency in [^201^Tl]thallium SPECT, with high specificity, its low sensitivity warrants a cautious clinical interpretation. Consequently, this threshold should not be utilized as an absolute, definitive criterion to completely omit further viability testing. Instead, it may serve as a potential gatekeeping parameter when considering further testing, reinforcing that regions with intermediate uptake still require downstream evaluation of viability status.

Although to the best of the authors’ knowledge and based on the available literature, there are no recent studies that directly compared [^201^Tl]thallium CZT SPECT with [^18^F]FDG PET for myocardial viability assessment, a recent meta-analysis compared [^18^F]FDG PET and CZT SPECT MPI and found good agreement for both modalities (16), suggesting comparability in terms of the technology of both modalities. A recent study by Sano et al. examined myocardial viability assessment using [^99m^Tc]technetium tetrofosmin or [^201^Tl]thallium CZT SPECT, with late gadolinium enhancement cardiac MRI as the reference standard, and found that CZT SPECT demonstrated high accuracy, with an AUC of 0.97 for identifying non-viable segments (17).

To contextualize our findings, it is valuable to compare our study with existing CZT SPECT and conventional SPECT MPI literature in terms of study design, reference standards, quantitative criteria, and clinical implications.

Although our retrospective study shares a similar observational design with recent CZT SPECT viability studies, the role of [^201^Tl]thallium CZT SPECT is fundamentally different. Sano et al. utilized late gadolinium enhancement cardiac MRI as the reference standard to validate the diagnostic accuracy of [^201^Tl]thallium and [^99m^Tc]technetium tetrofosmin CZT SPECT, while also following patients longitudinally after revascularization to determine functional recovery (17). In contrast, our study evaluated the comparative yield between resting/stress sestamibi SPECT MPI and [^201^Tl]thallium CZT SPECT in a cross-sectional setting. Here, [^201^Tl]thallium SPECT serves as the practical reference standard to identify possible viability within seemingly “fixed” defects on sestamibi SPECT MPI imaging.

Methodologically, our quantitative approach using software-derived segmental uptake scores aligns with established semiquantitative scoring systems while offering objective continuous measurements. Sano et al. employed a standard four-point visual defect scale (scores of 3 or 4 indicating non-viability). Our protocol used objective percentage tracer uptake thresholds, classifying uptake <50% as abnormal. This corresponds well with the American Society of Nuclear Cardiology guidelines (18), which define severe defects (score 3) as having a ≥50% reduction in uptake. This methodological concordance strengthens the comparability of our uptake thresholds with conventional visual scoring systems.

The clinical impact of the reclassification observed in our study extends prior conventional SPECT literature into the modern CZT era. In a study using Anger camera SPECT, Kong et al. demonstrated that 69% of patients with fixed sestamibi defects showed significant [^201^Tl]thallium redistribution, which strongly predicted future short- and intermediate-term cardiac events (19). By utilizing high-sensitivity CZT SPECT alongside objective uptake quantification, our study extends these findings by demonstrating that sestamibi tracer uptake must drop to very low levels (<20%) before non-reclassification can be predicted with high specificity.

Because Sano et al. demonstrated that [^201^Tl]thallium CZT SPECT correlates strongly with late gadolinium enhancement cardiac MRI and predicts functional recovery after revascularization, the identification of reclassified segments in our cohort is clinically meaningful. It indicates that even in patients displaying seemingly severe “fixed” defects on sestamibi SPECT MRI, the possibility of reclassification and thus possible viability and the potential for functional recovery may remain.

Consequently, relying solely on sestamibi SPECT MPI risks underestimating myocardial viability, even in segments with very severe uptake deficit. In clinical environments where gold-standard modalities like [^18^F]FDG PET or cardiac MRI are limited or unavailable, secondary evaluation with [^201^Tl]thallium CZT SPECT may therefore remain a viable alternative modality in these patients.

We acknowledge that this study is limited by its retrospective design and the lack of correlation with histological benchmarks, post-revascularization wall motion recovery, or alternative imaging modalities such as [^18^F]FDG PET or late gadolinium enhancement cardiac MRI. Consequently, the conversion of a segment from <50% to ≥50% maximum uptake on [^201^Tl]thallium SPECT should be interpreted strictly as a radiotracer diagnostic reclassification rather than definitive proof of functional recovery. However, our objective was not to validate [^201^Tl]thallium myocardial SPECT as an absolute gold standard, but rather to evaluate the real-world diagnostic interplay and reclassification rates between two standard protocols readily available on contemporary CZT hardware.

Moreover, the study design introduces an inherent structural selection bias, as [^201^Tl]thallium SPECT was performed exclusively in patients with preexisting fixed defects on initial rest/stress [^99m^Tc]Tc-sestamibi SPECT MPI. Consequently, the observed reclassification rates (48.0% at the segment level and 78.9% at the patient level) reflect the yield strictly within this preselected patient subgroup and may not be fully generalizable to a broader unselected population undergoing SPECT MPI.

There are also several additional methodological and statistical limitations. First, the ROC analysis used to examine the rest [^99mTc^]Tc-sestamibi uptake cutoff thresholds was exploratory in nature and derived from a single dataset without internal or external validation. Consequently, these quantitative cutoff values should be viewed as exploratory rather than validated rules. Second, image quality and segmentation were overseen by a single nuclear medicine specialist. Segmental myocardial uptake was determined using automated, quantitative software-derived uptake percentages (17-segment polar maps). Although these objective, uptake-based values minimize subjective interobserver variability, the lack of a formal multi-reader interobserver reproducibility analysis remains a study limitation.

The clinical justification for performing sequential radionuclide exams must be balanced against cumulative patient radiation exposure. In our protocol, patients received an estimated effective radiation dose of 9.3 mSv from the rest/stress sestamibi study. The subsequent thallium study which utilized a total of 2.5–4.0 mCi of [201Tl]thallium translated to approximately 13.0–20.7 mSv of additional effective radiation dose. This radiation burden should be considered when choosing [^201^Tl]thallium as an alternative modality in resource-constrained settings where access to established modalities such as [^18^F]FDG PET and CMR is restricted.

## 5 Conclusion

In patients presenting with fixed defects on routine rest/stress sestamibi SPECT MPI, the addition of dedicated thallium CZT SPECT provides incremental information, reclassifying nearly half of severely hypoperfused segments as having preserved uptake. Furthermore, while a resting sestamibi uptake of <20% demonstrated high specificity of 99% for non-reclassification, its low sensitivity of 12% indicates that its utility is restricted to a small subset of cases with severe defects on the initial sestamibi SPECT MPI. It should therefore be interpreted cautiously as an exploratory gatekeeping benchmark when considering downstream testing rather than as a rigid rule to omit further myocardial viability evaluation.

## Data Availability

The datasets presented in this study can be found in online repositories. The names of the repository/repositories and accession number(s) can be found below: Zenodo repository: https://doi.org/10.5281/zenodo.20556629.
