# Supplementary material for: Incremental value of dedicated [201Tl] cadmium-zinc-telluride thallium SPECT over rest/stress [99mTc] technetium sestamibi SPECT MPI: a comparative analysis of fixed perfusion defects
Source: Front Cardiovasc Med. 2026 Oct 2;13:1901315. doi: 10.3389/fcvm.2026.1901315 (PMC13672228; doi:10.3389/fcvm.2026.1901315)
Supplement: Supplementary file 1 [file Datasheet1.docx]

***Supplemental Table 1. Patient characteristics, with subgroup details.***

| **Characteristic** | **Whole cohort**  **(174 patients)** | | **Direct next day 24-hr [201Tl]thallium protocol (112 patients)** | | **Rest-redistribution [201Tl]thallium protocol (62 patients)** | |
| --- | --- | --- | --- | --- | --- | --- |
|  | **Value** |  | **Value** |  | **Value** |  |
| Age (years, means ± SD) | 65.0 | ± 13.6 | 64.5 | ± 14.4 | 65.8 | ± 12.0 |
| Sex |  |  |  |  |  |  |
| Male | 134 | 77.0% | 88 | 79% | 46 | 74% |
| Female | 40 | 23.0% | 24 | 21% | 16 | 26% |
| Body weight (kilograms, means ± SD) | 64.0 | ± 14.4 | 63.8 | ± 13.8 | 64.2 | ± 15.5 |
| Height (centimeters, means ± SD) | 162.1 | ± 7.0 | 162.3 | ± 6.8 | 161.8 | ± 7.3 |
| [^201^Tl]thallium myocardial SPECT results |  |  |  |  |  |  |
| All segments have ≥50% maximum uptake | 57 | 32.8% | 37 | 33.0% | 20 | 32.3% |
| At least one segment has <50% maximum uptake | 117 | 67.2% | 75 | 67.0% | 42 | 67.7% |

***Supplemental Table 2. Correlation and agreement of uptake scores between rest and stress [99mTc]Tc-sestamibi SPECT MPI with [201Tl]thallium myocardial SPECT, with subgroup details.***

| **Measure** | **Comparator with [^201^Tl] thallium myocardial SPECT** | **Whole cohort** | | | **Direct next day 24-hr [201Tl]thallium protocol** | | | **Rest-redistribution [201Tl]thallium protocol** | | |
| --- | --- | --- | --- | --- | --- | --- | --- | --- | --- | --- |
|  |  | **Value** | **95% CI.*** | **p-value** | **Value** | **95% CI.*** | **p-value** | **Value** | **95% CI.*** | **p-value** |
| Spearman’s rank correlation coefficient | Stress sestamibi SPECT MPI | 0.790 | 0.776 – 0.803 | < 0.001 | 0.782 | 0.763 – 0.799 | < 0.001 | 0.817 | 0.795 – 0.8357 | < 0.001 |
|  | Rest sestamibi SPECT MPI | 0.817 | 0.805 – 0.829 | < 0.001 | 0.812 | 0.796 – 0.826 | < 0.001 | 0.837 | 0.818 – 0.855 | < 0.001 |
| Intraclass correlation coefficient | Stress sestamibi SPECT MPI | 0.781 | 0.767 – 0.796 | < 0.001 | 0.769 | 0.750 – 0.787 | < 0.001 | 0.810 | 0.788 – 0.830 | < 0.001 |
|  | Rest sestamibi SPECT MPI | 0.812 | 0.800 – 0.824 | < 0.001 | 0.799 | 0.782 – 0.815 | < 0.001 | 0.840 | 0.821 – 0.857 | < 0.001 |
| Cohen’s kappa | Stress sestamibi SPECT MPI | 0.491 | 0.454 – 0.529 | < 0.001 | 0.415 | 0.464 – 0.513 | < 0.001 | 0.535 | 0.475 – 0.594 | < 0.001 |
|  | Rest sestamibi SPECT MPI | 0.550 | 0.512 - 0.588 | < 0.001 | 0.495 | 0.543 – 0.592 | < 0.001 | 0.560 | 0.500 – 0.620 | < 0.001 |

*CI., Confidence interval.

***Supplemental Table 3. Proportion of patients and myocardial segments which converted from < 50% maximum uptake in rest/stress SPECT MPI to ≥ 50% maximum uptake in thallium myocardial SPECT, with subgroup details.***

| **Analysis approach** | **thallium myocardial SPECT results** | **Whole cohort** | | **Direct next day 24-hr [201Tl]thallium protocol** | | **Rest-redistribution [201Tl]thallium protocol** | |
| --- | --- | --- | --- | --- | --- | --- | --- |
|  |  | **Number** | **Percent** | **Number** | **Percent** | **Number** | **Percent** |
| Segment-wise | Segments that remained < 50% maximum uptake | 305 | 52.0% | 178 | 51.1% | 127 | 53.1% |
|  | Segments that converted to ≥ 50% maximum uptake | 282 | 48.0% | 170 | 48.9% | 112 | 46.9% |
| Patient-wise | Patients whose results remained unchanged | 32 | 21.1% | 21 | 21.4% | 11 | 20.4% |
|  | Patients with at least one segment converted to ≥ 50% maximum uptake | 120 | 78.9% | 77 | 78.6% | 43 | 79.6% |
